# Outlier-Detection Methodology for Structural Identification Using Sparse Static Measurements

**DOI:** 10.3390/s18061702

**Published:** 2018-05-24

**Authors:** Marco Proverbio, Numa J. Bertola, Ian F. C. Smith

**Affiliations:** 1 ETH Zurich, Future Cities Laboratory, Singapore-ETH Centre, 1 CREATE Way, CREATE Tower, Singapore 138602, Singapore; numa.bertola@epfl.ch; 2 Applied Computing and Mechanics Laboratory (IMAC), School of Architecture, Civil and Environmental Engineering (ENAC), Swiss Federal Institute of Technology (EPFL), CH-1015 Lausanne, Switzerland; ian.smith@epfl.ch

**Keywords:** structural identification, model falsification, outlier detection, static measurements, bridge load tests, reserve capacity

## Abstract

The aim of structural identification is to provide accurate knowledge of the behaviour of existing structures. In most situations, finite-element models are updated using behaviour measurements and field observations. Error-domain model falsification (EDMF) is a multi-model approach that compares finite-element model predictions with sensor measurements while taking into account epistemic and stochastic uncertainties—including the systematic bias that is inherent in the assumptions behind structural models. Compared with alternative model-updating strategies such as residual minimization and traditional Bayesian methodologies, EDMF is easy-to-use for practising engineers and does not require precise knowledge of values for uncertainty correlations. However, wrong parameter identification and flawed extrapolation may result when undetected outliers occur in the dataset. Moreover, when datasets consist of a limited number of static measurements rather than continuous monitoring data, the existing signal-processing and statistics-based algorithms provide little support for outlier detection. This paper introduces a new model-population methodology for outlier detection that is based on the expected performance of the as-designed sensor network. Thus, suspicious measurements are identified even when few measurements, collected with a range of sensors, are available. The structural identification of a full-scale bridge in Exeter (UK) is used to demonstrate the applicability of the proposed methodology and to compare its performance with existing algorithms. The results show that outliers, capable of compromising EDMF accuracy, are detected. Moreover, a metric that separates the impact of powerful sensors from the effects of measurement outliers have been included in the framework. Finally, the impact of outlier occurrence on parameter identification and model extrapolation (for example, reserve capacity assessment) is evaluated.

## 1. Introduction

Sensing in the built environment has shown the potential to improve asset management by revealing intrinsic resources that can be exploited to extend the service life of infrastructure [1]. However, sensors on infrastructure often provide indirect information since effects, rather than causes, are measured. Physics-based models are necessary to convert this information into useful knowledge of as-built structure behaviour. Nonetheless, civil-engineering models involve uncertainties and systematic biases due to their conservative, rather than precise, objectives. Therefore, great care is required when measurements are used to improve the accuracy of model predictions, especially when the same models have been used for design.

In the field of structural identification, measurements are employed to update parameter values affecting structure behaviour. Residual minimization, also known as model-calibration, consists of adjusting model parameters to minimize the difference between predicted and measured values. This approach is the most common data-interpretation technique and has been a research topic for several decades [2,3,4]. However, several authors [5,6,7] have noted that while calibrated parameter values may be useful for interpolation, they are usually inappropriate for extrapolation. Unfortunately, extrapolation is needed for asset-management tasks such as the widening of bridge decks, retrofitting, and the comparison of competing future-proofing scenarios.

Bayesian model updating is the most common population-based structural-identification approach. This approach updates the initial knowledge in terms of probabilistic distributions of parameters by including the probabilistic distribution of measurement observations. The accurate identification of parameter values can be obtained using non-traditional implementations of Bayesian model updating [8]. However, methodologies that require deep knowledge of conditional probability and precise knowledge of statistical distributions, as well as their correlations, are not familiar and easy-to-use for practising engineers, who ultimately assume professional responsibility for decisions. Therefore, black-box approaches are not appropriate in such contexts. Other population-based methodologies, including falsification approaches, which sacrifice precision for accuracy, have provided accurate results when dealing with ill-posed problems and systematic modelling uncertainties [9]. 

Error-domain model falsification (EDMF) [10] is an engineering-oriented methodology that helps identify candidate models—models that are compatible with behaviour measurements—among an initial model population. EDMF provides parameter identification without requiring precise knowledge of levels of uncertainty correlations. Engineers simply define model uncertainties by providing upper and lower bounds for parameter values and model accuracy. First, the initial model population is generated; then, falsification methods are employed to detect and reject wrong models whose predictions are not compatible with measurements. Models that represent the real behaviour with a defined confidence are accepted and stored in the candidate model set (CMS).

The EDMF performance in identifying parameter values depends on factors such as the adopted sampling approach to generate the initial model population, the selection of relevant parameters, and the sensor configuration. An adaptive EDMF-compatible sampling approach that is able to outperform traditional sampling techniques has been recently proposed in Reference [11]. Some algorithms have been proposed to maximise the identification performance of sensor configurations by reducing the number of candidate models [12,13,14].

A basic hypothesis of all structural-identification methodologies is that measurement datasets do not include wrong data. Anomalous values in measurements, which are often called outliers, can occur due to faulty sensors or unexpected events during the monitoring process [15]. From a statistical point of view, there are several methods to identify outliers. If they are taken to be observations that are very far from other observations, data mining techniques can be employed for their detection [16,17]. However, outliers may seem to occur because of model deficiencies in the model classes [18]. 

In the field of damage detection, the presence of even a small number of wrong measurements or missing data reduced the performance of most algorithms [19,20]. Moreover, environmental variability and operational influence can affect model features in different ways, thus, leading to incorrect damage detection [21]. A comparison of methods to identify outliers and replace wrong measurement values in signal processing was proposed in Reference [22]. The solutions applicable to the measurement datasets affected by missing data can be found in References [23,24]. Additionally, a methodology that unifies data normalization and damage detection through the identification of measurement outliers has been proposed in Reference [25]. However, methodologies that are designed for detecting anomalies in continuous measurement contexts such as those described in References [26,27] have not been found to be suitable to examine datasets that consist of non-time-dependent measurements (for example, measurements of changes in stress, rotation, and displacement under static load testing). Such static measurements have been the most commonly used measurement strategies for large civil infrastructure since they are informative, they are easily comparable to code requirements, and they are the least costly. Effective support to analyse and validate sparse static measurements for outliers is currently unavailable.

In structural identification, the presence of outliers reduced the performance of current methods in terms of identification accuracy and prediction reliability. In Bayesian model updating, the classes of methods for outlier detection were proposed. The main two classes were based respectively on probabilistic measures such as posterior probability density function of errors [28] and L1 or Chi-square divergences [29]. Heavy-tailed likelihood functions such as Student’s t distribution or a combination of Normal and Student’s t distribution [30] have been employed for robust parametric estimations. Another class of methods treats outliers by assuming an outlier generation model, although, in practical applications, the information required to build such a model has often been unavailable [31].

Pasquier and Smith in [32] proposed an outlier-detection framework for EDMF, which is based on a sensitivity analysis of the CMS, with respect to sensor removal from the initial set. Model falsification was carried out iteratively while measurements provided by sensors were removed one at a time for any load case and the corresponding variations in CMS populations were noted. If anomalous high values of variation were obtained, then the measurement data was removed from the dataset. This framework represented only a semi-quantitative method for performing the outlier detection task as no rational definition of limits for CMS variations was proposed. As a result, sensors with the capability to falsify several model instances risked detection as outliers.

This paper presents a new outlier-detection framework that is compatible with population approaches such as EDMF. The proposed strategy is based on a metric used to evaluate the expected performance of sensor configurations that are often employed to optimize sensor placement. Additionally, a context metric that separates the impact of powerful sensors from the effects of measurement outliers has been included in the framework. The new approach is, therefore, suitable to analyse data sets that consist of sparse non-time-dependent measurements and overcomes limitations that characterise existing outlier-detection methodologies.

The remainder of the paper is organised as follows. Section 2 contains background information on EDMF and the proposed framework for outlier detection. In Section 3, the results of a full-scale case study are presented. Finally, the advantages and limitations of the proposed method are discussed.

## 2. Materials and Methods

### 2.1. Background—EDMF

Error-domain model falsification (EDMF) [10] is a recently developed methodology for structural identification in which the finite-element (FE) model predictions are compared with measurement data in order to identify plausible model instances of a parameterized model class. A model instance is generated by assigning unique combinations of parameter values to a model class g(·), which consists of an FE parametric model including characteristics such as material properties, geometry, boundary conditions, and actions.

Let $R_{i}$ be the real response of a structure—unknown in practice—at a sensor location i, and $y_{i}$ be the measured value at the same location. The model predictions at location i, $g_{i}\left(\theta \right)$, are generated by assigning a vector of parameter values θ to the selected FE model class. Model uncertainty $U_{i,g}$ and the measurement uncertainty $U_{i,y}$ are estimated and linked to the real behaviour using the following equation:

(1) $$g_{i}\left(\theta \right)+\;U_{i,g}=R_{i}=y_{i}+U_{i,y}\;\forall i\in \left\{1,\ldots ,n_{y}\right\},$$

where $n_{y}$ is the number of measurement locations. The terms in Equation (1) can be rearranged and the two sources of uncertainty ($U_{i,g}$ and $U_{i,y}$) can be merged in a unique term $U_{i,c}$, thus, leading to the following relationship:

(2) $$g_{i}\left(\theta \right)-y_{i}=U_{i,c}.$$

In Equation (2), the difference between a model prediction and a measured value at location i, is referred to as the residual $r_{i}=g_{i}\left(\theta \right)-y_{i}$.

Measurements errors $U_{y}$ includes sensor accuracy—based on the manufacturing specifications and site conditions—and the measurement repeatability that is usually estimated by conducting multiple series of tests on site. The model–class uncertainty source $U_{g}$, which is often dominant over $U_{y}$, is estimated using engineering judgment, technical literature, and local knowledge. Since a limited number of parameters can be sampled to generate the model class, an additional error—estimated using stochastic simulations—is often included in $U_{g}$. 

Plausible behaviour models are selected by falsifying those for which residuals exceeds the thresholds boundaries that are defined in the uncertainty domain (that is, the error domain). Being a falsification approach, EDMF initially requires that a set of model instances is generated by assigning parameter values to the model class. Then, the threshold bounds are defined at each sensor location as the shortest interval $\left[u_{low},u_{high}\right]$ that contains a probability equal to $\Phi_{d}^{1/n_{y}}$, using the following equation:

(3) $$\Phi_{d}^{1/n_{y}}=\int_{u_{i,low}}^{u_{i,high}}f_{U_{c,i}}\left(u_{c,i}\right)du_{c,i}\;\forall i\in \left\{1,\ldots ,n_{y}\right\},$$

where $f_{U_{c}}\left(u_{c}\right)$ is the combined probability density function at each sensor location, while the confidence level $\Phi_{d}$ is adjusted using the Sidák correction to take into account the simultaneous use of multiple measurements to falsify model instances.

Models for which residuals are within the threshold bounds ($u_{low},u_{high}$) at each sensor location are included in the candidate model set (CMS). The models for which residuals exceed these bounds, at one or more sensor locations, are falsified and, therefore, rejected.

When a candidate model set is identified, the prediction tasks involve using the CMS to assess the reserve capacity of the structure. Predictions $Q_{j}$ at locations j are given by

(4) $$Q_{j}=g_{j}\left(\theta^{\prime \prime }\right)+U_{j,g},$$

where $\theta^{\prime \prime }$ is a set of combinations of parameter values representing the CMS and $U_{g}$ is the model uncertainty. When all initial model instances generated are falsified, the entire model class is falsified. This means that no model is compatible with the observations given the current estimation of model and measurement uncertainties. Thus, it is usually a sign of incorrect assumptions in the model–class definition and uncertainty assumptions. Complete falsification helps avoid the wrong identification of parameter values and detects wrong initial assumptions, highlighting one of the main advantages of EDMF compared with other methodologies [5]. However, the wrong falsification of the entire CMS can occur because of the presence of outliers in the measurement data set.

The sensor configuration—designed according to the behaviour measurements to be collected—has a high sensitivity to the precision and accuracy of EDMF. The approach described in Reference [13] and extended in Reference [14], used simulated measurements to provide probabilistic estimations of the expected number of candidate models obtained with a sensor configuration. The aim was to find the sensor configuration that minimizes the expected number of candidate models. The simulated measurements are generated based on the model instances adding a random value taken from the combined uncertainties. Sensor locations were evaluated using respectively 95% and 50% quantiles of the expected candidate-model-set size. However, the procedure is computationally costly [33], because it requires the execution of the falsification procedure for a large number of simulated measurements and sensor locations. This issue has been acknowledged in References [34,35], where the expected identification performance is used as a metric to evaluate the information gain of a sensor configuration rather than being used as an objective function to be optimised.

### 2.2. Methodology

**Figure 1:**
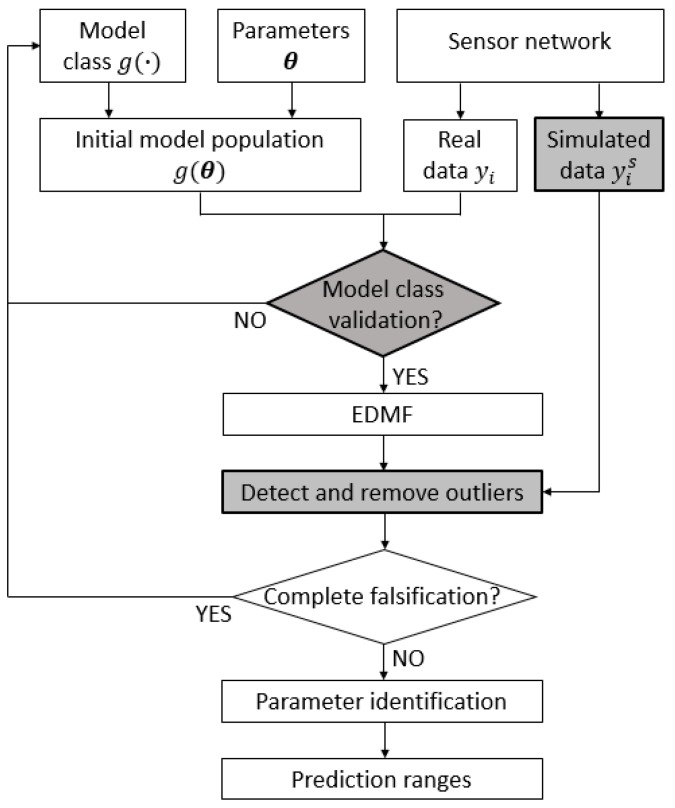
The general framework for the structural identification using error-domain model falsification (EDMF). The contributions of this paper are highlighted by the shaded boxes.

This paper proposes a new framework to improve the robustness of EDMF against the presence of anomalous values in measurement datasets and to detect flaws in the definition of the FE model classes. Figure 1 shows the general EDMF framework for structural identification, in which specific contributions introduced in this paper are highlighted by the shaded boxes.

The model–class validation is carried out by comparing predictions of the initial model population with measurements of real behaviour. This check is performed before updating the parameter values since the flawed model classes usually lead to wrong parameter identification. When an accurate model class is used, the measurement data can be compared with model predictions and EDMF identifies the ranges of the parameter values that explain the real behaviour. However, the presence of the outliers in the measurement datasets may lead to incorrect results. Outlier detection is particularly challenging when the measurement data consist of unique values collected under static conditions, rather than signals obtained from continuous monitoring. The proposed methodology takes advantage of the simulated measurements to compute the expected performance of a sensor network. Anomalous situations can be detected by comparing the expected and actual performance of (i) each sensor individually, and (ii) the entire sensor configuration. Sensors that are deemed to be suspicious are removed. Finally, the CMS is computed using only reliable measurements. The proposed model–class validation and outlier-detection methodology are described in detail in the next sections.

Interpolation tasks (that is, predicting at unmeasured locations) and, mostly, extrapolation tasks (that is, assessment of reserve capacity) represent the ultimate aims of structural identification. The extrapolation tasks are intrinsically more demanding since the fictitious parameter values do not compensate for model–class errors [9]. The outlier occurrence and inaccurate model classes can lead to wrong reserve-capacity assessments; thus, reiterating the importance of ensuring the robustness of identification methodologies.

#### 2.2.1. Model–Class Validation

**Figure 2:**
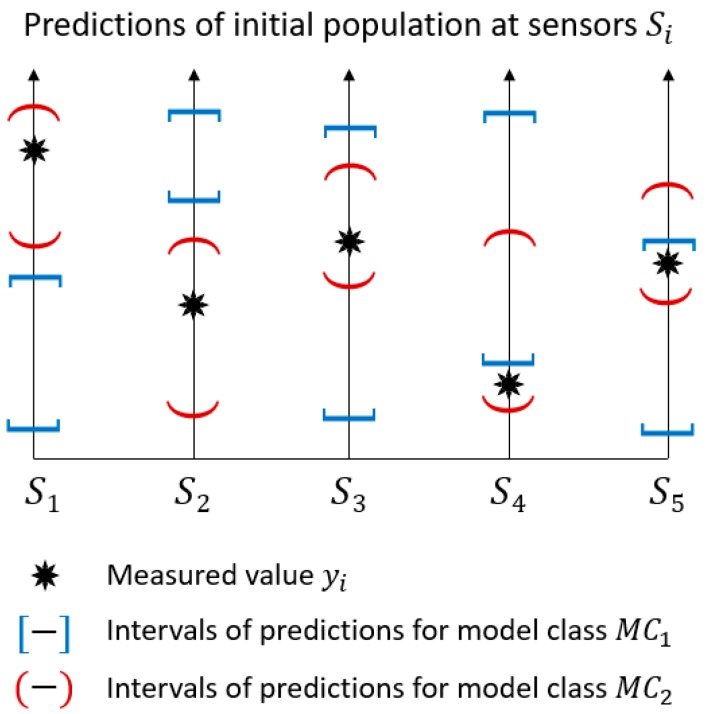
The model–class validation methodology. The model classes for which the prediction intervals of the initial population do not include measured values at several locations may reveal flaws in the model class definition, rather than the outliers in the measurement dataset.

Model–class accuracy is checked by comparing prediction ranges that are computed using the initial model population with measured values at each sensor location. A qualitative comparison between the two model classes, namely $MC_{1}$ and $MC_{2}$, is shown in Figure 2.

Each vertical axis represents a sensor and the prediction ranges are depicted using interval bounds. Measured values $y_{i}$ are included in the prediction intervals obtained using model class $MC_{2}$ for all locations i, while predictions of $MC_{1}$ do not include the measured values for sensor $S_{1}$, $S_{2}$, and $S_{4}$. As a result, $MC_{1}$ is unlikely to provide accurate explanations of the measured behaviour. In this situation, engineers should revise the model class assumptions, for example, through collecting further information during the inspection of the site. This iterative approach to structural identification is described in Reference [29]. 

However, the situation depicted in Figure 2 may have alternative explanations. For example, the measured value of sensor $S_{4}$ is close to the lower bound of the prediction ranges for both model classes. This suggests verifying that the initial ranges of behaviour parameters are sufficiently wide and that an appropriate sample density has been achieved. Alternatively, the measurements can be far from the prediction ranges due to the presence of many outliers in the dataset. The situation presumed in this paper involves a limited amount of sensors since outliers typically amount to less than 20% of the entire dataset [21].

#### 2.2.2. Outlier Detection

Unlike continuous monitoring in which a large amount of data is collected over time, datasets obtained during static tests often consist of a few measurements that are related to specific static configurations. Even when the same test is performed multiple times—usually to assess the measurement repeatability under site conditions—the amount of values collected from each measurement is insufficient to carry out statistical analyses. Therefore, anomaly detection cannot be performed by uniquely analysing the dataset.

**Figure 3:**
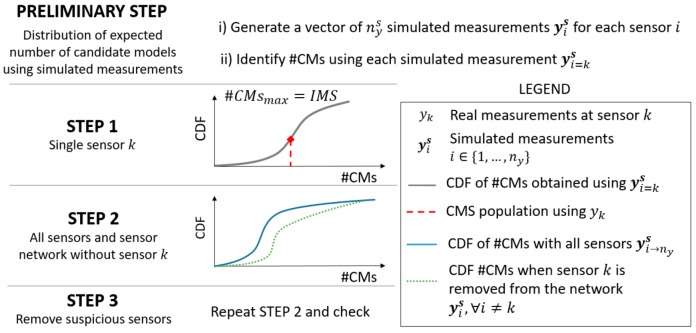
The outlier-detection steps.

Figure 3 outlines the framework that is proposed in this paper for outlier detection. In order to detect suspicious measurements, first, a vector of $n_{y}^{s}$ simulating measurements $y_{i}^{s}$ (that is, 100,000 measurements) is generated for each sensor location i by adding a random value of combined uncertainty $U_{i,c}$ to each model prediction $g_{i}\left(\theta \right)$ in Equation (2), according to Equation (5).

(5) $$y_{i}^{s}=g_{i}\left(\theta \right)-rand\left(U_{i,c}\right)\;\forall i\in \left\{1,\ldots ,n_{y}\right\}.$$

Then, using EDMF for each set of simulated measurements and the corresponding number of candidate models in the CMS (that is, the candidate-model-set population #CMs) is recorded. This number represents the expected dimension of the CMS population if a specific set of $y_{i}^{s}$ was used. Assuming that an accurate model class g(·) is used and no outlier affects the dataset, the distribution of the expected #CMs include the value that is obtained when the real measurement $y_{k}$ is used.

Given a sensor k, three steps should be performed to ensure that the measured value $y_{k}$ is plausible. In step 1, the cumulative density function (CDF) of #CMs, obtained using only the simulated measurement for this sensor ($y_{i=k}^{s}$), is plotted. The CDF is used to compute the cumulative probability to observe the CMS population given by $y_{k}$. A low probability value (for example, <5%) suggests that $y_{k}$ is a suspicious measurement. In such a case, step 2 should be performed. 

In step 2, two CDFs are computed: (i) one using the entire sensor network, and (ii) the second one using the network without sensor k, which is omitted. The area in between the two CDFs represents the uniqueness of information provided by sensor k and can be seen as the relative capacity of sensor k to falsify model instances. The smaller the area, the lower the improvement of the EDMF performance that results from including sensor k into the sensor configuration. When multiple sensors are affected by suspicious measurements, step 1 reveals the sensors that should be removed simultaneously before checking the sensor configuration again (step 3). 

**Figure 4:**
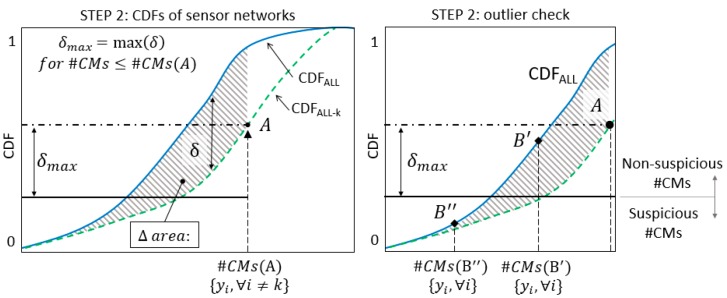
The outlier-detection procedure in step 2.

Figure 4 shows an example of the procedure to be completed in step 2. The CMS population #CMs(A) is obtained by performing falsification without sensor k and using real measurement data. The probability of observing a number of candidate models equal or greater than the #CMs, obtained when real measurements are employed and sensor k is omitted, is available from the graph (point A). The shaded area between the two CDFs for values of CMS populations lower than #CMs(A)—here referred to as Δ area—is identified and the maximum distance $\delta_{max}$, inside the Δ area, can be measured. The maximum distance $\delta_{max}$ is computed within the Δ area and it is not necessarily found at the same location of A. Finally, the maximum expected variation of probability that is associated with sensor k can be computed using $\delta_{max}$. This maximum expected variation is represented in Figure 4 by two horizontal lines (that is, the dash-dot line passing through A and the continuous line at distance equal to $\delta_{max}$). The maximum distance $\delta_{max}$ between the two CDFs can be a reasonable metric to define whether a certain variation in the #CMs, which results from the inclusion of an additional sensor k into the network, is plausible or suspicious—according to the expected performance of sensor k. 

When sensor k is included into the network, two scenarios are possible: (i) a reduction of #CMs—compared with #CMs(A)—is observed due to the additional information provided by sensor k, or (ii) no variation of #CMs is observed. In the latter case, sensor k does not contribute to improving the falsification performance of the network because of the redundancy of the current sensor configuration. When no variation of #CMs is obtained, there is no interest in evaluating the plausibility of measurements provided by sensor k since it does not affect the updating of the model. Alternatively, when the #CMs obtained using the entire network is lower than the previous case, the two situations depicted in Figure 4 by points $B^{\prime }$ and $B^{\prime \prime }$ can occur. If the reduction of #CMs is lower than the maximum expected variation of the CMS population—as it occurs for #CMs($B^{\prime }$)—the measurement provided by sensor k is deemed to be non-suspicious. Unexpected variations of #CMs, such as for #CMs($B^{\prime \prime }$), are considered suspicious; therefore, sensor k is treated as an outlier.

Finally, in step 3, the sensor that is deemed to be an outlier is removed from the sensor configuration and step 2 is performed iteratively until no suspicious data are found. Removing the suspicious sensors is an effective solution to avoid false-negative identification since the CMS obtained after excluding a sensor always includes the original CMS. Therefore, traditional outlier-correction strategies are not needed.

## 3. Results

### 3.1. Exeter Bridge Description

The Exeter Bascule Bridge (UK) has a single span of 17.3 m and was designed in 1972 to be lifted in order to allow the transit of boats along the canal. The light-weight deck, which consists of a series of flanked aluminium omega-shaped profiles, is connected to 18 secondary beams (type UB 533.210.82) that are bolted to two longitudinal girders (type UB 914.305.289). The bridge has a total width of about 8.2 m and carries the carriageway and a footway. The North-bank supports are hinges, while, on the South bank, the structure is simply supported. Two hydraulic jacks, which are activated during lifting manoeuvres, are connected to the two longitudinal girders on the North-bank side.

**Figure 5:**
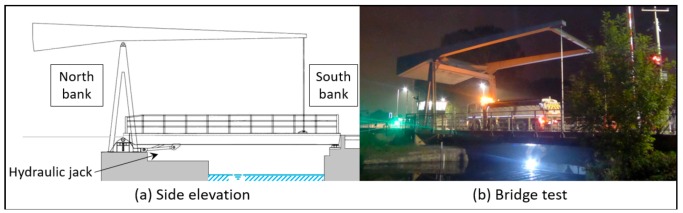
The Exeter Bascule Bridge: (**a**) side elevation; (**b**) static load test.

A static load test was performed to collect the mid-span vertical displacements and strain measurements at several locations. Figure 5 shows the side elevation and a view of the bridge during the load test. Additional information about the Exeter Bascule Bridge can be found in Reference [36].

### 3.2. Parameters and Modelling Uncertainties

Three parameters that influence the structural behaviour are selected for model updating, namely: the equivalent Young’s modulus of the aluminium deck ($\theta_{1}$), the rotational stiffness of the North-bank hinges ($\theta_{2}$), and the axial stiffness of the hydraulic jacks ($\theta_{3}$). The initial intervals for each parameter are presented in Table 1. The bridge deck consists of aluminium planks with an omega-shaped cross-section bolted to secondary beams. In the FE model, the deck has been modelled using a plate with the equivalent thickness simply supported by secondary beams. Considering this simplification, a uniform distribution with sufficiently large bounds was conservatively chosen to describe the initial knowledge of this parameter. The values for the rotational stiffness cover the full range from a constrained to a pinned support, in order to include potential effects due to the corrosion of bearings. The axial stiffness of hydraulic jacks is used to simulate their contribution as additional load-carrying supports. The lower bound for the axial stiffness is equivalent to assuming the two girders simply supported at the abutments. The upper bound corresponds to the introduction of a semi-rigid support at jack connections. 

An initial population consisting of 3000 instances is generated from the uniform distribution of each parameter value using Latin hypercube sampling. Uncertainties associated with the FE model class are defined as percentages that are applied to the mean values of the initial-model-set predictions. The forms and magnitudes of the estimated uncertainties are reported in Table 2.

The main source of uncertainty due to FE model simplifications is not symmetric. All secondary beams are perfectly fixed to the longitudinal girders, instead of having perfectly pinned connections. Therefore, the FE model is actually stiffer than the real structure, thus, justifying the increment of the model predictions up to 20%. However, assumptions such as the omissions of non-structural elements (for example, barriers) could have the opposite effect, leading to a more flexible behaviour than the real one. The latter omission has a smaller influence on the bending behaviour, thus, the model uncertainty range is asymmetric. The bounds for this source of uncertainty have been defined using conservative engineering judgments, as recommended in Reference [37].

Typical uncertainties that relate to the FE method such as the mesh refinement and additional uncertainties are estimated according to the technical literature. The mesh-refinement uncertainty has been quantified through a convergence analysis, by increasing the mesh density until the model response converged asymptotically and the prediction variations were lower than 1%. An analogous practice is described in Reference [38]. Additional uncertainties help account for accidental omissions and for the phenomena that, when taken individually, have a negligible impact. Finally, the uncertainties have been initially reduced by site inspection, which also involved the checking of element geometry. Values similar to those reported in Table 2 have been previously employed in studies concerning full-scale bridges [32,39]. 

### 3.3. Sensor Configuration

**Figure 6:**
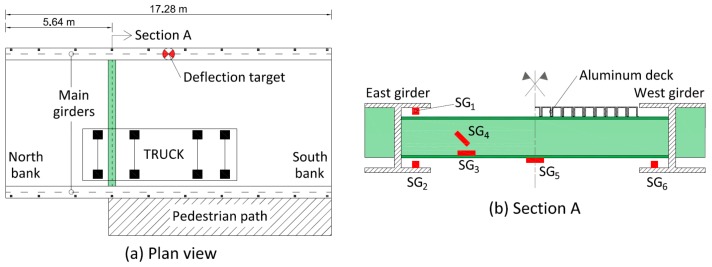
The Exeter Bascule Bridge: (**a**) the plan view including the sensor locations and the truck position; (**b**) the locations of the strain gauges on the main girders and the elevation of the instrumented secondary beam.

The adopted sensor configuration consists of six strain gauges that are glued to the main girders and a selected secondary beam. Additionally, a deflection target was installed on the East girder at mid-span and a precision camera was used to record the vertical displacements. The sensor configuration and the truck position are depicted in Figure 6.

Uncertainties associated with the sensor configuration are reported in Table 3. The uncertainty magnitudes are described as absolute values or percentages of measured values. The sensor accuracy is provided by the manufacturer specifications while the measurement repeatability is estimated by performing multiple measurements under site conditions. For strain gauges, the uncertainty also arises from the imperfect alignment of gauges with respect to the longitudinal axes of girders and secondary beams, which often results in the underestimation of real stresses. The imperfect bonding between the strain gauges and elements may also influence strain measurements. These errors are assessed using engineering judgments and the conservative ranges are selected for the uniform uncertainty distribution. Further details on the uncertainty assessment can be found in Reference [40].

### 3.4. Results for Model–Class Validation

**Figure 7:**
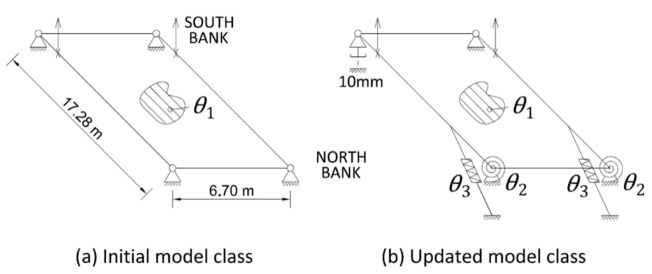
The model–class definitions: (**a**) the initial model class; (**b**) the updated model class.

In order to perform the model–class validation, the two model classes depicted in Figure 7 are generated. The initial model class involves typical design assumptions idealising the bridge as a frame that is simply supported by four non-friction bearing devices. Assuming the structure geometry and the elastic properties of steel to be well-known, the equivalent Young’s modulus of the aluminium deck ($\theta_{1}$) is the only parameter to be identified.

The updated model class includes friction connections on the North-bank side of the bridge, the two hydraulic jacks that are used for lifting, and the presence of a 10-mm gap between the base plates of the main girders and the abutment at the South-West support. The presence of the gap was observed during the visual inspection of the structure, confirming the iterative nature of structural identification.

The model–class validation described in Section 2.2.1 is performed and the results are presented in Table 4 and Table 5.

When the initial model class is employed (Table 4), the prediction ranges of the initial population include the measured value at only one sensor location (that is, $SG_{5}$) out of seven. Additionally, at an few locations (for example, $SG_{1},SG_{2}$, and $SG_{6}$), the measurements are extremely far from the prediction ranges. On the contrary, the observed behaviour of the bridge is captured by the updated model class, which is intrinsically more detailed than the initial one. In Table 5, all the measurements belong to the initial prediction ranges, which include the combined uncertainties.

### 3.5. Results for Outlier Detection

The detection of suspicious values in the measurement datasets is carried out according to the two-step methodology presented in Section 2.2.2.

**Figure 8:**
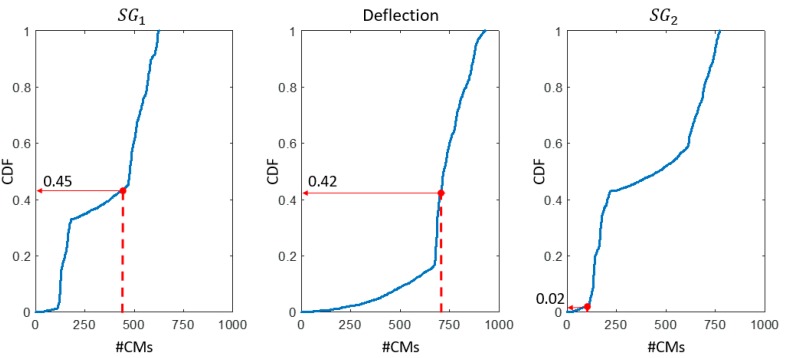
The outlier-detection methodology showing: Step 1—for three sensors ($SG_{1}$, the deflection camera, and $SG_{2}$), the cumulative density function (CDF) of #CMs, obtained using simulated measurements (solid lines). The values of CMS population (#CMs) using real measurements are indicated by the dashed lines and the corresponding probability values from the simulated measurements are determined.

The analysis of each sensor is performed individually in step 1. In Figure 8, the CDFs of the CMS populations computed using simulated measurements for the three most effective sensors ($SG_{1}$, the deflection camera, and $SG_{2}$), are plotted. Then, the cumulative probability of observing the #CMs obtained using the real measurement of each sensor (identified as a dot of the CDF) is computed. A probability of 2% is obtained for sensor $SG_{2}$, while the other sensors show probability values around 40%. The high falsification performance of sensor $SG_{2}$ compared with the average of the sensor network, suggests that this sensor provides suspicious data. The results for the remaining four sensors ($SG_{3}$ to $SG_{6}$) are similar to those shown in Figure 8 for sensor $SG_{1}$ and the deflection camera.

Although removing $SG_{2}$ would be a simple solution, at the current stage, no information is available on the relative falsification performance of sensor $SG_{2}$. Therefore, further investigation is necessary to avoid the risk of wrongly excluding effective sensors.

**Figure 9:**
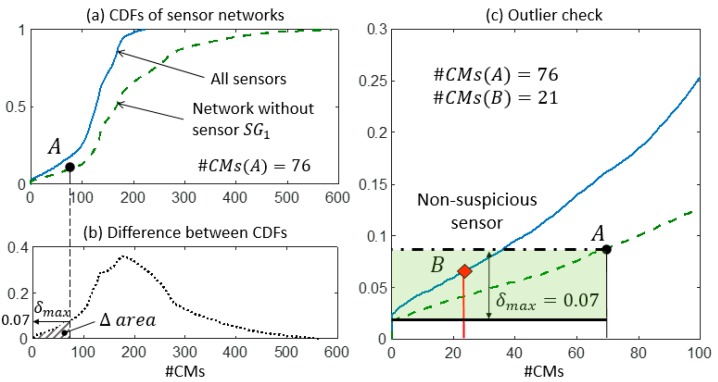
The outlier detection: step 2. (**a**) CDFs of the expected #CMs using all sensors and the network without $SG_{1}$. (**b**) $\delta_{max}$ is computed as the maximum distance between the two CDFs in the Δ area. (**c**) The detail of the two CDFs and the outlier check.

**Figure 10:**
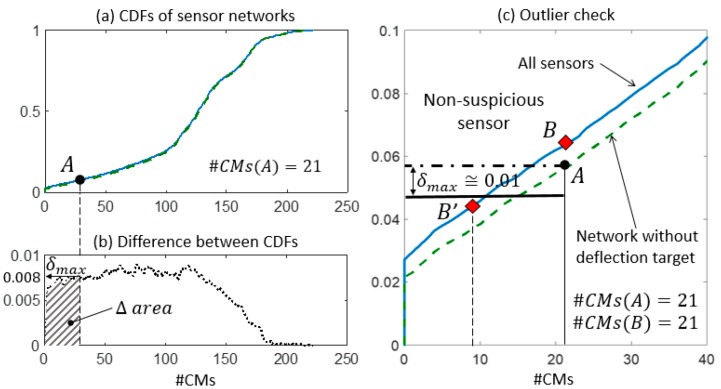
The outlier detection: step 2. (**a**) CDFs of the expected #CMs using all sensors and the network without the deflection measurement. (**b**) $\delta_{max}$ is computed as the maximum distance between the two CDFs in the Δ area. (**c**) The detail of the two CDFs and the outlier check.

**Figure 11:**
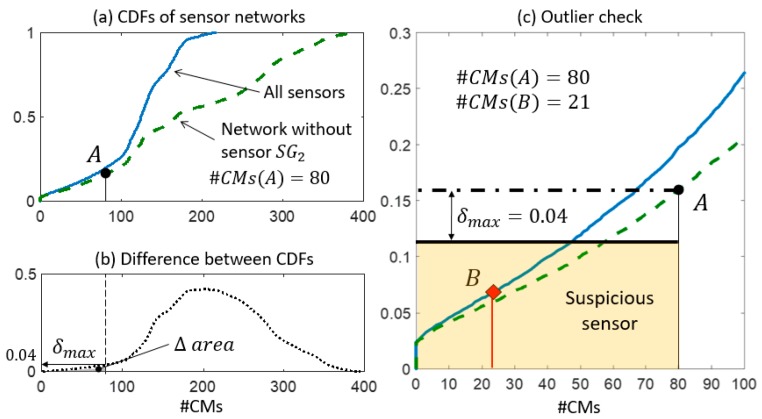
The outlier detection: step 2. (**a**) CDFs of the expected #CMs using all sensors and the network without $SG_{2}$. (**b**) $\delta_{max}$ is computed as the maximum distance between the two CDFs in the Δ area. (**c**) The detail of the two CDFs and the outlier check.

In step 2, the expected performance of each sensor is assessed and compared with the actual values of falsification performance. In Figure 9a, Figure 10a, and Figure 11a, two CDFs are shown: one (continuous line) using the entire sensor network, and the second one (dashed line) using the network without sensor k. To help compute the maximum distance $\delta_{max}$, the difference between the CDFs is represented in function of the expected #CMs using dotted lines and dashed areas above the x-axis in Figure 9b, Figure 10b, and Figure 11b. Figure 9c, Figure 10c, and Figure 11c show, in greater detail, the portions of interest of the CDFs. The CMS populations are computed using real measurements; first, while sensor k is omitted #CMs(A), then, using the entire network #CMs(B). The values of the cumulative probability for each condition (points A and B) are available from the corresponding CDFs. Finally, $\delta_{max}$ is used as a metric to define whether the variation in the #CMs—the horizontal distance between A and B—is plausible or suspicious.

In Figure 9c, the contribution of sensor $SG_{1}$ to the falsification performance of the network is shown by the reduction of #CMs from 76 to 21. This variation can be explained by the expected performance of $SG_{1}$, which is estimated as a reduction of the cumulative probability of 7% ($\delta_{max}=0.07)$. Since the observed reduction is lower than the expected one, the measurement provided by $SG_{1}$ is deemed to be plausible.

Similarly, in Figure 10c, the falsification performance of the deflection measurement is analysed. However, this sensor does not contribute to the falsification since no variation of #CMs is observed and #CMs(A) is equal to #CMs(B). Therefore, the information provided by the deflection measurement is redundant with respect to the current sensor configuration. Since the CDF computed using all sensors is always above the CDF obtained when a sensor is removed from the network, point B is located above point A in Figure 10c. In this situation, the computation of $\delta_{max}$ is superfluous, since no outliers can be detected using the presented methodology. When a redundant sensor is removed from the network, the corresponding CDF is almost coincident with the CDF that is computed using the entire network and low values of $\delta_{max}$ are possible. However, since no variation of #CMs occurs, the redundant sensors are not detected as outliers. For B to become a suspicious sensor, a variation of the candidate model set using real measurements would need to be approximately 50% of the number of candidate models of A (see point B’ in Figure 10). This illustrates the robustness of the method when the difference between the two CDFs is small.

The falsification performance of $SG_{2}$ is analysed in Figure 11. In step 1, Sensor $SG_{2}$ was detected as a possible source of outliers because of its high falsification performance compared with the average of the network. Figure 11c shows the reduction from #CMs(A) = 80 to #CMs(B) = 21 that occurs when $SG_{2}$ is included in the network. Such a variation cannot be justified by the reduction of the cumulative probability by 4% ($\delta_{max}=0.04)$ since point B lies outside the $\delta_{max}$ band. Therefore, the anomalous measurement provided by $SG_{2}$ should be treated as an outlier.

It is worth noting that a large variation of #CMs is not always connected to anomalous measurements. For example, #CMs variations for sensor $SG_{1}$ and $SG_{2}$ are similar; however, the expected reduction $\delta_{max}$ for $SG_{1}$ is almost twice the reduction for $SG_{2}$. As a conclusion, the metric introduced by the expected reduction $\delta_{max}$ provides a rational support in evaluating the CMS variations.

**Figure 12:**
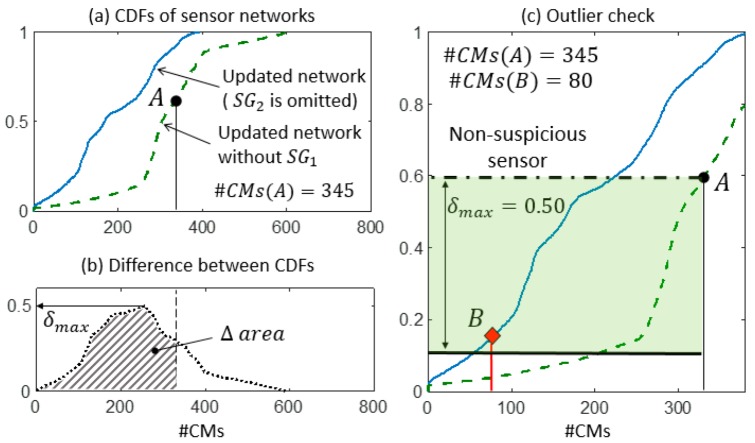
The updated sensor network—without the suspicious sensor $SG_{2}$—is checked. (**a**) CDFs of the expected #CMs using the updated network without $SG_{2}$. (**b**) $\delta_{max}$ is computed as the maximum distance between the two CDFs in the Δ area. (**c**) The detail of the two CDFs and the outlier check.

Finally, in step 3, the sensor network is updated by removing sensor $SG_{2}$ and step 2 is performed again to ensure that no outlier remains. Figure 12 shows, for example, the outlier-detection check for sensor $SG_{1}$ when the updated sensor network is employed. Since no sensor provides suspicious variations of #CMs, the updated sensor network is considered to be reliable and the CMS can be computed.

**Figure 13:**
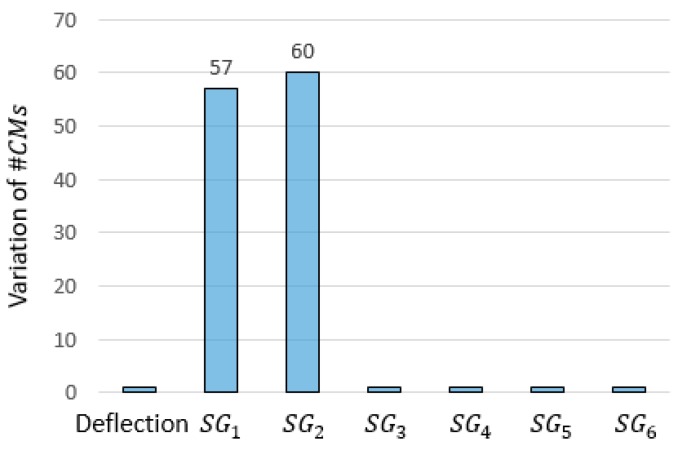
The variation of #CMs when one sensor at a time is removed and falsification is carried out iteratively. High variations reveal suspicious measurements, according to Reference [32].

For comparison, Figure 13 reports results that could be obtained by implementing the outlier-detection strategy proposed in Reference [32]. The approach proposed by Pasquier et al. requires that the falsification is carried out iteratively while measurements provided by sensors are removed one at a time. The corresponding variations of #CMs are recorded and, in case of anomalous high values of variation being obtained, the measurement is removed from the dataset. However, when two or more sensors produce high variations of #CMs, it is hard to distinguish the powerful sensors from those that are affected by the outliers. On the contrary, the methodology proposed here clearly identifies the anomalous data source in sensor $SG_{2}$.

Since sensor $SG_{2}$ is considered to be an outlier, in the remainder of this paper, it is excluded from the sensor configuration.

### 3.6. Detection of Simulated Outliers

Simulated outliers are used in this section to test the proposed methodology. Table 6 presents a range of noteworthy scenarios in which outliers have been generated by applying percentage variations to real measurements or by replacing measured values with wrong data.

In all the scenarios, the proposed methodology is able to detect the simulated outliers. In scenario 2, a reduction of 20% of the true measurement leads to the complete falsification of the model class, while in scenario 5, 8 candidate models are found despite the fact that the $SG_{1}$ measurement increased by about 4 times its original value. The outliers that cause complete falsification (#CMs=0) can be detected using the model–class validation presented in Section 2.2.1.

**Figure 14:**
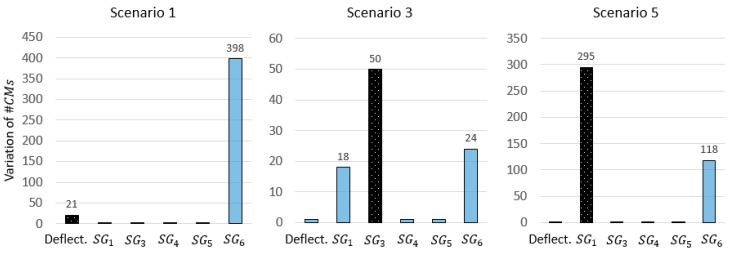
The variation of #CMs when one sensor at a time is removed and falsification is carried out iteratively. The dark bar refers to the sensor where the outliers are simulated as described in Table 6.

When the variations of #CMs that result from simulated outliers are analysed using the methodology proposed in Reference [32], several issues are encountered. Figure 14 shows the results corresponding to scenarios 1,3, and 5 in Table 6. Although the two sources of outliers show the highest variation in scenarios 2 and 3, no guidance is provided regarding the other sensors that show high variations. As a result, engineers may conservatively opt to remove all sensors that show high variations, leading to a drastic reduction of the global identification performance.

In scenario 1, sensor $SG_{6}$ clearly exhibits the highest variation of #CMs, when the outlier is simulated in the deflection measurement. This results in the wrong identification of the outlier source. Again, the variation of the CMS population alone is not a reliable metric to evaluate the plausibility of the measurement data.

## 4. Discussion

The presence of outliers in the measurement datasets can reduce the accuracy of the structural-identification methodologies such as EDMF. Table 7 compares the identification results obtained when the outliers replace the true measurements (scenarios 3 and 5) with the no-outlier scenario. For example, in scenario 3, the presence of an outlier at sensor $SG_{3}$ results in the wrong falsification of plausible low values of $\theta_{1}$. Additionally, the outlier simulated in scenario 5 leads to a wrong identification of the values for parameter $\theta_{3}$ (all values fall outside of the ranges found when there is no outlier). The presence of the outliers can lead to unpredictable variations of identified ranges for parameters and the number of candidate models. As a consequence, wrong extrapolations can result when the outliers are not identified and removed.

The reserve capacity of an existing structure can be defined, for a defined limit state, as the ratio between the design load—given by codes—and the as-built maximum loads—computed using models. In the model class, the test loads are replaced by design load configurations, in which all the relevant safety factors are applied. The serviceability limit state (SLS) of stress control is investigated for the Exeter Bascule bridge by checking that under characteristic design loads, the maximum Von Mises stress in each element is lower than the yield strength ($f_{y}=345\;\mathrm{MPa}$). A detailed description of the procedure for the reserve-capacity assessment is available in Reference [41]. 

Table 8 provides a comparison of the serviceability of reserve-capacity assessments. The outlier simulated in scenario 3 provides a small variation of the reserve capacity (around 1%). However, in scenario 5, the unidentified outlier results in an overestimation of the reserve capacity by more than 10%. During extrapolation, which is the ultimate aim of structural identification, the consequences of outlier occurrence are unpredictable. In conclusion, removing the outliers from the dataset is crucial to ensure the accurate parameter identification and reliable model extrapolation.

In this paper, it is assumed that the outliers usually amount to less than 20% of the entire dataset [22]. Consequently, a unique outlier was expected from the adopted sensor configuration, consisting of 7 sensors. Multiple outliers can occur when larger sensor networks are employed. If two or more sensors are deemed to be suspicious when step 1 is performed, they should be temporarily removed in step 2, to avoid the risk that they compensate each other. However, the identification of several outliers in the dataset may result from the adoption of flawed model classes rather than anomalous datasets. Both situations should be investigated.

The detected outliers are removed from the dataset and falsification is carried out again until no suspicious values are found. The proposed methodology predicts the consequences of removing sensors that provide plausible results from the sensor configuration. Therefore, the combined uncertainties rather than measurement uncertainties are added to the model predictions to generate simulated measurements. This ensures that real measurements are included in the ranges of the simulated measurements generated using accurate model classes. Consequently, the plausible measurements are not likely to be wrongly detected as outliers (false positive). However, if a false positive occurs and the sensor is removed, the resulting CMS becomes larger, thus, including the true model that would have been identified using sensors that were incorrectly removed.

The framework presented in this paper compares the expected and current performance of the sensor configuration by mapping the effects that outliers have on the CMS. Therefore, data sets that consist of sparse static measurements can be validated. A context metric ($\delta_{max}$) is used to evaluate the effects of removing suspicious sensors from the current configuration, thus, allowing to distinguish between powerful sensors from outliers. Finally, this approach outperforms existing methodologies that have been previously applied to structural identification based on the EDMF approach.

The results presented in Section 3.4 and Section 3.5 refer to the real measurements collected on site. In Section 3.6, the five scenarios are designed to avoid presenting the trivial case, in which complete falsification results from the presence of outliers, several times. This situation is presented only in scenario 2. The outliers in redundant sensors are likely to be detected since $\delta_{max}$ is small, while the limitations related to the lack of redundancy in the sensor configurations are discussed below. Therefore, the remaining four scenarios focus on the most powerful sensors available in the network. Scenario 3 shows a case in which the algorithm proposed by Pasquier and Smith [32] leads to a wrong detection. Scenario 5 was defined to discuss the effects of the undetected outliers on parameter identification and reserve-capacity assessment. Scenario 4 was selected to demonstrate that the outliers in sensor $SG_{1}$ can be detected even when their magnitude is not as extreme as assumed in Scenario 5. Given the assumed uncertainty magnitudes, measurement variations lower than 20% may not be considered suspicious by engineers.

The following limitations of the proposed framework are recognised. The sampling technique adopted to generate the model population and the assessment of uncertainties influence identification results and the generation of simulated measurements. Additionally, alternative approaches may be employed to generate simulated measurements. Moreover, accurate parameter identification and successful outlier detection are possible only when the reasonable model classes are adopted. Model–class features and model uncertainties should always be verified through visual inspection and iterative model–class updating when new information becomes available.

The environmental conditions under which the test is performed may affect the values of the identified parameters. In the EDMF methodology, the environmental variability can be accounted for by explicit modelling and the measurement of environmental effects, including additional sources of uncertainties and by repeating the test multiple times in various conditions. The environmental variability should not affect the outlier detection methodology since the analysis is based on the variation of the CMS population when a sensor is removed from the network. While different test conditions may provide varying CMS populations for points A and B, the relative variation of the CMS population between the two points when a sensor is removed should not be affected.

Partial sensor redundancy is crucial to ensuring the robustness of the sensor configurations [42,43]. When very few sensors are employed, $\delta_{max}$ increases to the point of accepting very large variations in the number of candidate models. Consequently, outlier detection is unlikely since the relative importance of each sensor determines the falsification performance. Therefore, suspicious measurements may be accepted when there are very small numbers of sensors. Finally, understandably, the likelihood of successfully detecting outliers depends on the magnitudes of uncertainty. Outlier measurements close to the true value may be considered non-suspicious when modelling and measurement uncertainties are high.

## 5. Conclusions

Population methods for structural identification are not robust when there are outliers in the measurements. The proposed methodology, based on the expected performance of sensor identification, helps reveal the outliers that compromise the accuracy of data interpretation. Compared with previous algorithms, suspicious measurements are more efficiently checked using the information provided by the entire sensor configuration. A metric that separates the impact of powerful sensors from the effects of measurement outliers provides a useful tool for asset managers.

## Figures and Tables

**Table 1:** The initial ranges of the parameters to be identified.

Parameters	Initial Intervals
$\theta_{1}$—Equivalent Young’s modulus of aluminium deck (GPa)	[60; 80]
$\theta_{2}$—Rotational stiffness of bearing devices (log(Nmm/rad))	[8; 12]
$\theta_{3}$—Axial stiffness of hydraulic jacks (log(Nmm))	[3; 5]

**Table 2:** The model–class uncertainty estimation.

Uncertainty Source	Uncertainty Form	Uncertainty Magnitude
FE model simplification (%)	Uniform	−5%; +20%
Mesh refinement (%)	Uniform	−1%; +1%
Additional (%)	Uniform	−2%; +2%

**Table 3:** The measurement uncertainty estimation.

Uncertainty Source	Uncertainty Form	Uncertainty Magnitude
Sensor accuracy		
Camera (mm)	Uniform	−0.1; +0.1
Strain gauges (με)	Uniform	−2; +2
Measurement repeatability		
Camera (%)	Gaussian	μ=0; σ=1
Strain gauges (%)	Gaussian	μ=0; σ=1.5
Sensor installation		
Strain gauges (%)	Uniform	−2%; +2%

**Table 4:** The initial model–class validation.

Prediction Intervals	Deflection (mm)	$SG_{1}$ (με)	$SG_{2}$ (με)	$SG_{3}$ (με)	$SG_{4}$ (με)	$SG_{5}$ (με)	$SG_{6}$ (με)
Minimum	7.7	23.2	111.5	0.8	12.1	48.5	227.9
Maximum	9.9	28.6	119.7	20.8	16.4	78.1	255.7
Measurement	6.8	4.3	1.8	21.7	17.7	73.8	83.5
Validation	✗	✗	✗	✗	✗	**✓**	✗

✓: The prediction intervals include the measurement. ✗: the prediction intervals do not include the measurement.

**Table 5:** The updated model–class validation.

Prediction Intervals	Deflection (mm)	$SG_{1}$ (με)	$SG_{2}$ (με)	$SG_{3}$ (με)	$SG_{4}$ (με)	$SG_{5}$ (με)	$SG_{6}$ (με)
Minimum	1.0	−2.4	−2.7	0.5	12.2	48.8	−20.8
Maximum	13.2	71.3	107.0	23.5	18.0	80.5	267.9
Measurement	6.8	4.3	1.8	21.7	17.7	73.8	83.5
Validation	**✓**	**✓**	**✓**	**✓**	**✓**	**✓**	**✓**

✓: The prediction intervals include the measurement. ✗: the prediction intervals do not include the measurement.

**Table 6:** The simulated outliers that replace the true measurement.

Scenario	Sensor	True Measurement	Simulated Outlier	#CMSs	Detection
1	Deflection	6.79 mm	+25%	56	**✓**
2	Deflection	6.79 mm	−20%	0	**✓**
3	$SG_{3}$	21.7 με	−20%	28	**✓**
4	$SG_{1}$	4.25 με	1.3 με	4	**✓**
5	$SG_{1}$	4.25 με	18 με	8	**✓**

✓ Indicates that the simulated outlier has been identified.

**Table 7:** The updated values of parameters in the presence of undetected outliers.

Parameter	Scenario: No Outlier	Scenario: 3	Scenario: 5
$\theta_{1}\left(GPa\right)$	(60.2; 79.8)	(71.5; 79.8)	(63.8; 74.8)
$\theta_{2}\left(\log\left(\mathrm{Nmm}/\mathrm{rad}\right)\right)$	(8.08; 11.94)	(8.29; 11.91)	(9.01; 10.16)
$\theta_{3}\left(\log\left(\mathrm{Nmm}\right)\right)$	(4.28; 4.35)	(4.29; 4.34)	(4.46; 4.47)

**Table 8:** The serviceability-limit-state reserve-capacity assessments in the presence of undetected outliers.

Scenario	SLS Reserve Capacity
No outlier	2.09
Scenario 3	2.07
Scenario 5	2.31
